# Dietary total antioxidant capacity as a preventive factor against depression in climacteric women

**DOI:** 10.1590/1980-57642018dn13-030007

**Published:** 2019

**Authors:** Natiani Gonçalves de Oliveira, Iranice Taís Teixeira, Heloísa Theodoro, Catia Santos Branco

**Affiliations:** 1 Universidade de Caxias do Sul Área do Conhecimento de Ciências da Vida Caxias do SulRS Brasil Área do Conhecimento de Ciências da Vida, Universidade de Caxias do Sul, Caxias do Sul, RS, Brasil.; 2 Universidade de Caxias do Sul Instituto de Biotecnologia Laboratório de Estresse Oxidativo e Antioxidantes Caxias do SulRS Brasil Laboratório de Estresse Oxidativo e Antioxidantes, Instituto de Biotecnologia, Universidade de Caxias do Sul, Caxias do Sul, RS, Brasil.

**Keywords:** mood disorders, depression, oxidative stress, antioxidants, polyphenols, transtornos de humor, depressão, estresse oxidativo, antioxidantes, polifenóis

## Abstract

Depression is characterized by physical or psychological distress and in many cases can lead to suicide.

**Objective:**

to assess the prevalence of depression and its possible relationship with dietary total antioxidant capacity (DTAC) and nutritional parameters in climacteric women participating in an extension university program in a Southern Brazilian city.

**Methods:**

data were obtained through questionnaires and anthropometric measurements. Diet was assessed using a 24-hour dietary recall. The Beck Depression Inventory (BDI) was used to assess the intensity of symptoms of depression.

**Results:**

DTAC of the population ranged from 435.60 to 4502.62 mg VCE/day. Among the most consumed antioxidant food/beverages, coffee ranked highest. Polyphenols were found to be directly linked to the antioxidant capacity of fresh foods (r=0.905; p=0.0001). Prevalence of depression in the population was 44%, and depressed women had lower intake levels of polyphenols (p=0.022; Cohen's d=0.80), and vitamin B6 (p=0.038; Cohen's d=0.65), vitamin A (p=0.044; Cohen's d =0.63), and vitamin C (p=0.050; Cohen's d =0.61). There was a significant negative correlation between BDI scores and polyphenol intake (r=-0.700; p=0.002).

**Conclusion:**

these results may contribute to a better understanding of the recommended dietary antioxidant intake as an adjuvant for preventing depression in women.

## RESUMO

Depressão é caracterizada por causar sofrimento físico ou psicológico e em muitos casos pode levar ao suicídio.

**Objetivo:**

avaliar a prevalência de depressão e sua possível relação com a capacidade antioxidante total da dieta (DTAC) e parâmetros nutricionais em mulheres climatéricas participantes de um programa universitário de extensão em uma cidade do sul do Brasil.

**Métodos:**

os dados foram obtidos por meio de questionários e medidas antropométricas. A dieta foi avaliada através de um recordatório alimentar de 24 horas. O Inventário de Depressão de Beck (BDI) foi usado para avaliar a intensidade dos sintomas de depressão.

**Resultados:**

a DTAC da população variou de 435,60 a 4502,62 mg VCE/dia. Entre os alimentos/bebidas antioxidantes mais ingeridos, o café destacou-se em primeiro. Os polifenóis mostraram estar diretamente ligados à capacidade antioxidante dos alimentos in natura (r=0,905; p=0,0001). A prevalência de depressão na população foi de 44%, e as mulheres depressivas apresentaram menores níveis de ingestão de polifenóis (p=0,022; Cohen's d=0,80) e vitaminas B6 (p=0,038; Cohen's d=0,65), A (p=0,044; Cohen's d=0,63) e C (p=0,050; Cohen's d=0,61). Houve uma correlação negativa significativa entre os escores do BDI e a ingestão de polifenóis (r=-0,700; p=0,002).

**Conclusão:**

estes resultados podem contribuir para uma melhor compreensão da ingestão dietética recomendada de antioxidantes como adjuvante na prevenção da depressão feminina.

Depression is characterized by distress, either physical or psychological; resulting from a complex interaction of social, psychological and biological factors, and in many cases can lead to suicide.^1^^,^^2^ There is a growing number of cases of depression worldwide. Between 2005 and 2015, there was an increase in depression cases of 18%; currently, there are 322 million people with this mental illness.^3^ Brazil has the second highest incidence of depression in the Americas, with a rate of 5.8%, behind only the United States of America, which has a 5.9% rate. In Brazil, the Southern region has the highest prevalence of depression in the country. The World Health Organization predicts that depression will be the main global health issue by 2030.

Regarding the disease's prevalence, women are the most affected, with two female cases diagnosed for every male case.^3^ One of the reasons that lead women to experience depression is hormonal change, in which the climacteric period is particularly important.^4^^-^6 Changes in estrogen secretion modify the levels of noradrenaline and serotonin in the brain.^7^ Therefore, the classic symptoms of depression, such as irritability, melancholy, mood and emotional lability, manifest.^5^ Apart from hypoestrogenism, other factors can contribute to the development of depression in women. One of these factors is the nutrient deficiency that occurs throughout life, especially with respect to some amino acids, vitamins and minerals.^8^^-^11 Moreover, it has been demonstrated that depression is linked to a decrease in circulating antioxidants.^12^^-^15

One of the most important classes of natural antioxidants are polyphenols, chemicals widely found in the plant food matrix. The most prevalent dietary phenolics are flavonoids, non-flavonoids, and stilbenes.^16^ These micronutrients are secondary plant metabolites that exhibit a broad spectrum of pharmacological activities, including neuroprotection^17^^,^^18^ and antidepressant action.^19^^,^^20^ Previous evidence shows that antioxidants present in foods from natural sources can influence brain dynamics and functioning through a preventive and/or therapeutic effect elicited by these compounds.^10^^,^^13^^,^^21^^,^^22^

While dietary phytochemicals promote an important either preventive or restorative effect in several chronic diseases, their role in depression needs further investigation. Furthermore, information about the intake of antioxidants by women is scarce. Therefore, this study aimed to evaluate the possible relationship between dietary antioxidant capacity, polyphenol intake and depression in climacteric women from the Southern region of Brazil.

## METHODS

This study was approved by the Ethics Committee for Research in Humans of the Universidade de Caxias do Sul under permit number 2.420.632; 07/12/2017. All procedures performed in studies involving human participants were in accordance with the ethical standards of the institutional research committee and with the 1975 Helsinki declaration and its later amendments or comparable ethical standards. Informed consent was obtained from all individual participants included in this study. Forty-one women aged between 50 and 69 years, participating in the UCS Senior extension program, were included. Exclusion criteria were: women who were taking a dietary supplement or currently undergoing chemotherapy. Participants answered questionnaires while supervised by the researchers. The first questionnaire collected data on demographic characteristics, physical activity (engagement in any type of exercise for at least 30 min), diagnosis of depression (patients previously diagnosed by a psychiatrist according to the DSM-IV), and antidepressant medication use. The second questionnaire evaluated the intensity of depression symptoms, measured using the Beck Depression Inventory (BDI), classified as: normal (0-9), mild (10-15), mild to moderate (16-19), moderate to severe (20-29), or severe (30-63).^23^ Information about the diet of the volunteers was obtained using a 24-hour dietary recall. Data were analyzed by the Diet box^®^ Software (Program for Nutritional Assessment professional version 2015, Brazil). For calculation of dietary total antioxidant capacity (DTAC), women were instructed to recall all known antioxidant foods consumed during the previous day. DTAC was calculated according to the antioxidant capacity of each food/beverage multiplied by the amount consumed per day.^24^ Results were expressed as mg equivalents of vitamin C per day (mg VCE/d).

Participants were submitted to an anthropometric measurement (weight, height and waist circumference) evaluation. Waist circumference was assessed according to the World Health Organization criteria.^25^ Body mass index (BMI) was classified according to the (WHO) for women aged between 50 and 60 years^26^ and to the Pan American Health Organization for women over 60 years old.^27^ In addition to the anthropometric measurements, participants' blood pressure was measured.

Statistical analyses were performed using SPSS software version 22.0 for Windows (SPSS Inc., Chicago, IL). Categorical variables were expressed as absolute and relative frequencies and the continuous variables as mean±standard error. Quantitative data were transformed using log for normalization purposes. Student's *t*-test for independent samples was used to compare the means of nutritional measurements between the depressed and non-depressed group. Effect size was calculated using Cohen's *d* on normalized data. Possible associations were analyzed by using Spearman's correlation analysis. Results were considered statistically significant for p-value ≤0.05.

## RESULTS

Demographic variables, lifestyle and nutritional aspects are shown in Table 1. Of the participants, 61% were aged 61-68 years, 32% had higher education and 32% postgraduate level. In relation to family income, 37% had an income of R$ 3,060-7,650 and 34% R$ 7,650-15,300 (Brazilian currency). Regarding the lifestyle and anthropometric indicators, 88% engaged in physical activity, 54% had optimal blood pressure (systolic pressure <120 mmHg). Among the volunteers, 54% had normal weight and 24% were overweight. Although obese women were a minority (22%), 58% of the participants had a waist circumference >88 cm.

**Table 1:** Description of sociodemographic characteristics, nutritional status and lifestyle of the volunteers (n=41).

Variable	Frequency (N)	Percentage (%)
**Age**		
• 50-55 years	5	12.2
• 56-60 years	11	26.8
• 61-68 years	25	61.0
**Educational level**		
• Incomplete Primary education	2	4.9
• Complete Primary education	2	4.9
• Complete Secondary Education	11	26.8
• Complete College/University	13	31.7
• Complete Postgraduate course	13	31.7
Family income*		
• < R$1,020	1	2.4
• R$1,020 - R$3,060	5	12.2
• R$3,060 - R$7,650	15	36.6
• R$7,650 - R$15,300	14	34.1
• > R$15,300	6	14.6
**Weekly physical activity**		
• Yes	36	87.8
• No	5	12.2
Blood pressure categories**		
• Optimal	22	53.7
• Normal	7	17.1
• High normal	4	9.8
• Grade 1 hypertension	2	4.9
• Grade 2 hypertension	4	9.8
• Grade 3 hypertension	2	4.9
Nutritional status (kg/m^2^)***		
• Normal	22	53.7
• Overweight	10	24.4
• Obesity	9	22
**Waist circumference (cm)**		
• <80	5	12.2
• 80-88	12	29.3
• >88	24	58.5

* Brazilian currency;

** According to the ESC/ESH Guidelines for the management of arterial hypertension^28^;

*** For women aged between 50 and 60 years, the classification used was: < 18.5 kg/m^2^ underweight, 18.5-24.9 kg/m^2^ normal weight, 25.0-29.9 kg/m^2^ overweight and >30 kg/m^2^ obesity.^26^ For women over 60 years old, the classification used was: <23 kg/m^2^ underweight, 23-28 kg/m^2^ normal weight, 28-30kg/m^2^ overweight and >30 kg/m^2^ obesity.^27^

**Figure 1:**
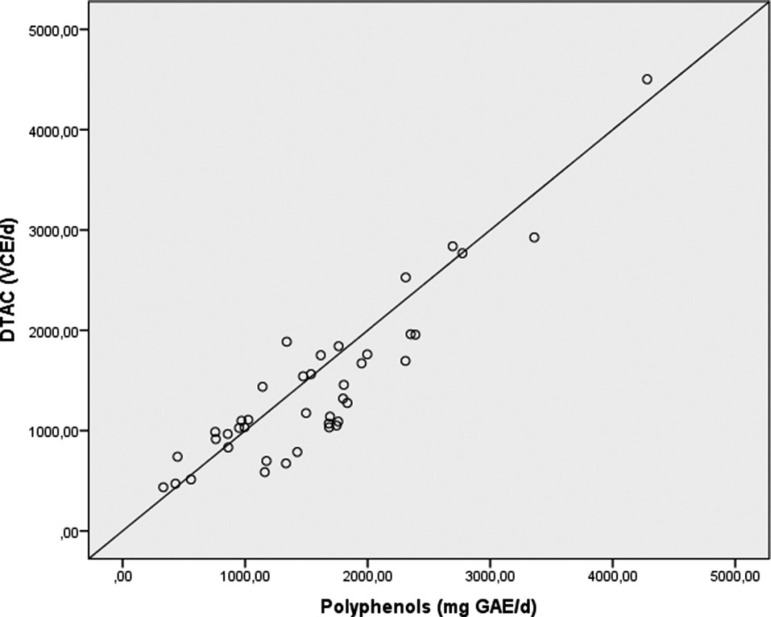
Spearman correlation between dietary total antioxidant capacity (DTAC) and total polyphenol content of the participant’s diet.

Intake of dietary polyphenols of the participants ranged from 332.03 to 4,281.15 mg GAE/d, while DTAC ranged from 435.60 to 4,502.62 mg VCE/d. These two variables had a strong positive correlation (r=0.905; p=0.0001; Figure 1), indicating that polyphenols are predominantly responsible for the antioxidant capacity of fresh foods. In addition, DTAC was also positively associated with family income (r=0.342, p=0.029) and educational level (r=0.498, p=0.001), suggesting that women with higher income and educational levels are more judicious in their food choices.

**Figure 2:**
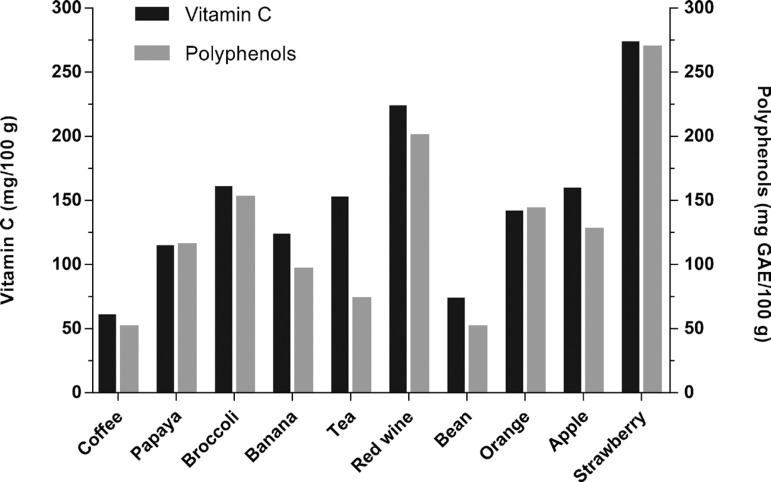
Vitamin C and total polyphenol content of the ten foods and drinks that most contributed to increasing the dietary total antioxidant capacity (DTAC) of the participant’s diet.

Figure 2 shows the ten foods and beverages that most contributed to raising the DTAC of the participants, as well as the respective amounts of vitamin C and phenolic content. The percentage contribution of each food/beverage was: coffee (11.9%), papaya (7.3%), broccoli (6.9%), bananas (6.5%), tea (*Camellia sinensis*) (6.3%), red wine (5.7%), beans (5.4%), oranges (5%), apples (4.1%), and strawberries (3.4%).

Among the 41 women evaluated, 18 presented depression (44%). Of these, 29% were taking antidepressant medication. Regarding the intensity of depressive symptoms, around 32% had mild intensity, followed by severe with 7.3% (Table 2). Antidepressant medications most reported by depressed participants were fluoxetine, paroxetine, sertraline, and escitalopram.

**Table 2:** Prevalence of depression, use of antidepressant medication, and intensity of depressive symptoms among the volunteers (n=41).

	Frequency (N)	Percentage (%)
**Depression**		
• Yes	18	43.9
• No	23	56.1
**Uses of antidepressant medication**		
• Yes	12	29.3
• No	6	14.6
Beck's inventory*		
• Mild	13	31.7
• Moderate	2	4.9
• Severe	3	7.3

* intensity of depressive symptoms.

With respect to the dietary characteristics and nutrient intake of these women (Table 3), intake levels of polyphenols (p=0.022) and vitamin B6 (p=0.038), vitamin A (p=0.044) and vitamin C (p=0.050) were lower in the depressed group, where the strongest effect was for polyphenols, followed by vitamins B6, A and C. Moreover, a negative correlation was found between severity of depressive symptoms and polyphenol intake levels (r=-0.700; p=0.002; Figure 3). Although not statistically significant, DTAC was found to be low in the depressed group (p=0.079; Cohen's *d*=0.60). No differences were found for macronutrient (carbohydrate, protein, and lipids) or mineral intake between the depressed and non-depressed groups.

**Table 3:** Dietary and nutrient intake characteristics of the volunteers (n=41).

	Depressed group (n=18)	Non-depressed group (n=23)	P-value	Effect size^#^
DTAC (mgVCE)	1189.52±132.04	1694.34±237.96	0.079	0.60
Polyphenols (mgGAE)	1261.92±130.68	1852.31±189.84	0.022	0.80
Total energy intake (Kcal)	1171.53±92.90	1309.91±74.54	0.247	0.36
Protein (% TEI)	17.96±0.80	20.53±1.79	0.242	0.37
Carbohydrate (% TEI)	49.85±3.46	52.01±2.71	0.621	0.16
Fat (% TEI)	29.18±2.31	27.46±1.87	0.563	0.18
Total fiber (% TEI)	18.69±2.37	21.54±1.72	0.327	0.31
Iron (mg)	7.73±0.90	8.22±0.71	0.671	0.14
Magnesium (mg)	205.49±23.03	238.98±14.84	0.211	0.40
Manganese (mg)	17.46±11.23	18.26±7.66	0.952	0.02
Zinc (mg)	6.74±0.71	7.77±0.61	0.275	0.35
Selenium (µg)	73.87±17.19	70.31±8.48	0.844	0.06
Vitamin A (µg)	515.88±109.10	878.51±183.57	0.044	0.63
Vitamin B1 (mg)	0.80±0.13	0.96±0.09	0.315	0.32
Vitamin B2 (mg)	1.14±0.12	1.33±0.11	0.264	0.35
Vitamin B3 (mg)	10.82±2.13	14.31±2.12	0.259	0.36
Vitamin B6 (mg)	1.09±0.12	1.45±0.13	0.038	0.65
Vitamin B9 (µg)	239.99±51.52	203.98±17.09	0.470	0.23
Vitamin B12 (µg)	2.15±0.42	2.57±0.41	0.488	0.22
Vitamin C (mg)	98.83±27.45	127.15±16.52	0.050	0.61
Vitamin D (µg)	2.16±0.62	2.96±0.80	0.457	0.24
Vitamin E (mg)	4.44±0.60	5.76±0.77	0.204	0.40

Data were normalized prior to analyses; however, raw data (mean±standard error) are presented.

# Calculated using Cohens's d on normalized data for 0.2-0.5 small effect size; 0.5-0.8 medium effect size and >0.8 large effect size. DTAC: Dietary total antioxidant capacity. VCE: vitamin C equivalents; GAE: gallic acid equivalents. Kcal: kilocalorie; TEI: total energy intake.

**Figure 3:**
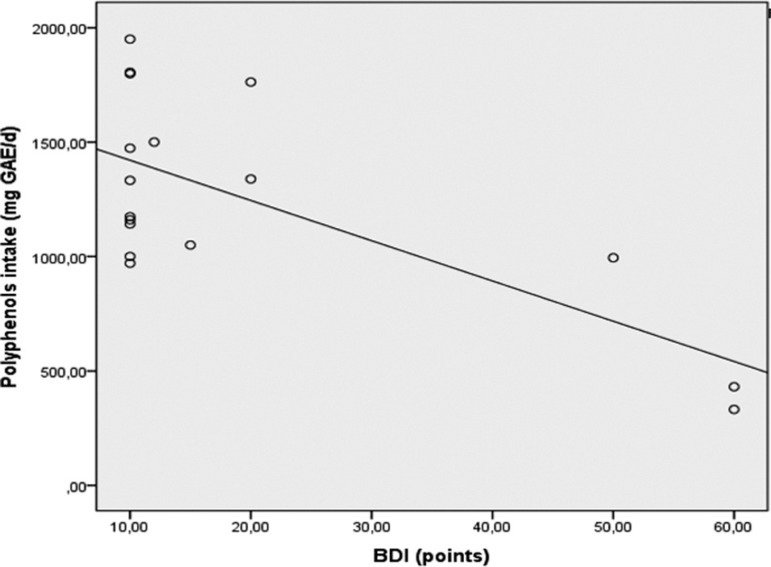
Spearman correlation between Beck Depression Inventory (BDI) score *versus* polyphenol intake in depressed women.

## DISCUSSION

This cross-sectional observational study reported the effects of dietary patterns in climacteric depressed women. Our findings indicate that climacteric depressed women exhibit lower intake of polyphenols.

Regarding the characteristics of the sample, this study showed that the volunteers had a waist circumference > 88 cm, which potentially increases the risk of metabolic complications, such as insulin resistance, hyperinsulinemia, impaired glucose tolerance, type 2 diabetes mellitus, dyslipidemia, visceral obesity^29^ and also brain disorders.^30^ Metabolic diseases can be avoided by following a low-calorie diet. The diet can be further improved by the ingestion of polyphenols,^29^ thus ameliorating systemic and central redox status.

Taken together, coffee, tea, and wine were the beverages responsible for 24% of the entire DTAC estimated. These drinks/foods have a significant content of vitamin C and/or phenolic compounds. Our findings are in accordance with the results obtained by Zuanazzi et al.,^31^ who also found high coffee intake by the climacteric female population. Coffee (*Coffea arabica* L.) is a polyphenol-rich beverage, which exhibits an expressive free radical scavenger ability. This capacity is important for attenuation of the body's redox imbalance, which is associated with the occurrence of several chronic diseases, such as neuropsychiatric disorders. Recently, coffee consumption was reported as a preventive factor for risk of depression in a systematic review and a dose-response meta-analysis of observational studies.^32^ In addition to coffee, broccoli stands out as the third most consumed antioxidant food. Broccoli contains sulforaphane, a compound that is able to reduce neuroinflammation observed in chronic and recurrent depression.^33^ These foods are also sources of tryptophan, an essential amino acid, the only precursor of serotonin (5-hydroxytryptamine, 5-HT). The balance between dietary intake of tryptophan and its removal from plasma determines plasma tryptophan concentration, and ultimately its bioavailability for the brain.^34^ 5-HT concentrations are linked to mood and perceptions of pain, wherein very low levels generate depressive and even suicidal behavior.^35^ Adequate intake of tryptophan and other micronutrients, such as magnesium, B-complex vitamins and vitamin D involved in the synthesis of 5-HT is essential for the prevention of its depletion.^11^^,^^34^ There is evidence showing that some micronutrients could even enhance the effects of selective serotonin reuptake inhibitors (SSRIs),^13^^,^^22^ which are the first line of pharmacologic treatment for depression.

Vitamins A (retinoic acid) and C (ascorbic acid) are recognized antioxidants that are able to control metabolic redox status. According to previous data, patients with depressive disorder commonly have vitamin C deficiency. This finding was reported by Bajpai et al.^15^ who showed that depressed patients have significantly decreased serum vitamin C levels compared to healthy individuals. Regarding vitamin C supplementation, Pullar et al.^36^ demonstrated that high levels of vitamin C in plasma were associated with improved overall mood in tertiary-level students aged 18 to 35 years. Moreover, in the study conducted by Gautam et al.,^37^ supplementation with vitamins C, A and E (α-tocopherol) for a period of 6 weeks resulted in a significant reduction in depression intensity scores, along with improvement in anxiety control among adults. Vitamin B6 has action on the central nervous system and, in the present study, lower intake of this vitamin by depressed women was found. In line with our findings, another study demonstrated that the intake of B-complex vitamins, particularly B6, is associated with a lower prevalence of depressive symptoms in early adolescence.^38^ Kim et al.^39^ also reported a negative correlation between intake of vitamin B6, C and ß-carotene (pre-vitamin A) and depression in adolescent girls, findings corroborated by our data. Along with vitamins, polyphenol intake was also low in depressed women. Furthermore, the severity of depressive symptoms was inversely correlated with polyphenol levels, suggesting that increased intake of these compounds in the diet could attenuate depressive symptoms. Although there are few studies in the literature about polyphenols intake by depressed women, Godos et al.^14^ showed that higher intake of flavonoids may be inversely associated with depressive symptoms in adults.

Although plasmatic total antioxidant capacity (PTAC) was not measured in the present study, it has been shown that DTAC is responsible for PTAC in women.^31^^,^^40^ Taken together, our data show that climacteric depressed women have a low intake of polyphenols and vitamins, molecules able to control levels of endogenous oxidative stress. While further studies are needed to conclusively confirm these associations, the present study can contribute to new dietary therapeutic strategies for this group of individuals, which has a high prevalence of depression compared to the general population.

When interpreting the study results, some limitations of our methods should be taken into account. First, dietary patterns were evaluated using a self-administered diet history questionnaire, which could lead to a subjective self-analysis. Nevertheless, given that a dietician guided the participants, we believe the results accurately reflect real life. Secondly, attention was focused on dietary patterns and serum biochemical parameters were not assessed, where the exact dose ranges needed to exert protective effects have yet to be determined. Lastly, this study evaluated a small sample and should be replicated in larger studies.

Although the exact mechanisms underlying the association between dietary intake of polyphenols/vitamins and depressive symptoms are unclear, the present study is the first to report the influence of dietary habits on the occurrence of depression in climacteric women. We found that depressed women had a lower intake of polyphenols and vitamins A, B6 and C in their diets. Moreover, our data revealed a trend toward decreasing DTAC levels in depressed women. Further studies involving large sample populations are needed to determine the cause-and-effect relationship between DTAC and depression in longitudinal studies.
